# Sexual counselling and sexual therapy in chemsex users in an NGO in spain

**DOI:** 10.1192/j.eurpsy.2021.1472

**Published:** 2021-08-13

**Authors:** J. Curto Ramos, I. Azqueta, M.T. Heredia Soriano, J.F. Cabrera Solano, L. Ibarguchi

**Affiliations:** 1 Chemsex Program “sexo, Drogas Y Tú”, Apoyo Positivo, madrid, Spain; 2 Mental Health Service, University Hospital La Paz, MADRID, Spain

**Keywords:** chemsex, NPS, sexuality

## Abstract

**Introduction:**

The intentional use of drugs before or during sexual intercourse (chemsex) is a phenomenon of special importance in the MSM (men who have sex with men) population due to its impact on mental, physical and sexual health. Sexual health issues related to chemsex practice have been described such as difficulties in achieving sober sex, erectile dysfunction or problems with sexual desire.

**Objectives:**

To describe the sexual health interventions (including sexual counselling and sexual therapy) for patients with chemsex practices in the NGO Apoyo Positivo in Madrid. We describe the main sexual problems.

**Methods:**

Descriptive analysis.

**Results:**

The main sexual problems were dissatisfaction in sexual intercourse without substance and difficulties with sexual desire activation (70%); compulsive sexual behaviour (70%), difficulties with sexual orientation and non normative gender expression, difficulties in erection (34%), premature ejaculation (7%) and delayed ejaculation (10%).

**Conclusions:**

Chemsex is a phenomenon that needs a multidisciplinary approach and mental and sexual health must be taken into account. “Sexo, Drogas y Tu” is a model of collaborative approach which is a pioneering intervention developed by an NGO in Spain.

